# Complete genome sequence of and proposal of *Thermofilum uzonense* sp. nov. a novel hyperthermophilic crenarchaeon and emended description of the genus *Thermofilum*

**DOI:** 10.1186/s40793-015-0105-y

**Published:** 2015-12-09

**Authors:** Stepan V. Toshchakov, Aleksei A. Korzhenkov, Nazar I. Samarov, Ilia O. Mazunin, Oleg I. Mozhey, Ilya S. Shmyr, Ksenia S. Derbikova, Evgeny A. Taranov, Irina N. Dominova, Elizaveta A. Bonch-Osmolovskaya, Maxim V. Patrushev, Olga A. Podosokorskaya, Ilya V. Kublanov

**Affiliations:** Immanuel Kant Baltic Federal University, Kaliningrad, Russian Federation; Pirogov Russian National Research Medical University, Moscow, Russian Federation; Winogradsky Institute of Microbiology, Research Center for Biotechnology Russian Academy of Sciences, Moscow, Russian Federation

**Keywords:** *Thermofilum*, *Crenarchaeota*, hyperthermophile, Kamchatka, Phylogeny, Polysaccharides hydrolysis

## Abstract

A strain of a hyperthermophilic filamentous archaeon was isolated from a sample of Kamchatka hot spring sediment. Isolate 1807-2 grew optimally at 85 °C, pH 6.0-6.5, the parameters being close to those at the sampling site. 16S rRNA gene sequence analysis placed the novel isolate in the crenarchaeal genus *Thermofilum*; *Thermofilum pendens* was its closest valid relative (95.7 % of sequence identity). Strain 1807-2 grew organothrophically using polysaccharides (starch and glucomannan), yeast extract or peptone as substrates. The addition of other crenarchaea culture broth filtrates was obligatory required for growth and could not be replaced by the addition of these organisms’ cell wall fractions, as it was described for *T. pendens*. The genome of strain 1807-2 was sequenced using Illumina and PGM technologies. The average nucleotide identities between genome of strain 1807-2 and *T. pendens* strain HRK 5^T^ and “*T. adornatus*” strain 1910b were 85 and 82 %, respectively. On the basis of 16S rRNA gene sequence phylogeny, ANI calculations and phenotypic differences we propose a novel species *Thermofilum uzonense* with the type strain 1807-2^T^ (= DSM 28062^T^ = JCM 19810^T^). Project information and genome sequence was deposited in Genbank under IDs PRJNA262459 and CP009961, respectively.

**Electronic supplementary material:**

The online version of this article (doi:10.1186/s40793-015-0105-y) contains supplementary material, which is available to authorized users.

## Introduction

Currently, the archaeal phylum *Crenarchaeota* comprises five orders: *Thermoproteales**,**Sulfolobales**,**Desulfurococcales**,**Acidilobales**and**Fervidicoccales* [1]. *Thermoproteales* representatives are characterized as rod-shaped extreme thermophiles and hyperthermophiles, growing either auto- or heterotrophically, using different redox pairs to gain energy as well as performing fermentation of organic substrates [2]. The order consists of two families: *Thermoproteaceae* [3], and *Thermofilaceae* [4]; the second one is a deeply branching lineage, consisting so far only one validly published genus and species, *Thermofilum pendens* isolated from a solfataric hot spring in Iceland [5] and characterized as an anaerobic hyperthermophilic, moderately acidophilic chemoorganotrophic archaeon utilizing peptides as the energy source and sulfur as the electron acceptor. Its growth is obligatory dependent on *Thermoproteus tenax* polar lipid fraction. Other *T. pendens* strains were isolated from solfataras of Yellowstone National Park (USA) and Vulcano Island (Italy) [4]. Notably, one of them described non-validly as a separate species “*Thermofilum librum*” has 100 % 16S rRNA gene sequence identity with *T. pendens*, but does not require the addition of other organisms’ cell components [6]. Recently, another species “*Thermofilum adornatus*” strain 1910b was isolated from a black mud pit (Kamchatka, Russia) and its genome was sequenced [7].

Here we describe another *Thermofilum* strain 1807-2, report its genomic sequence, what allow us to propose a novel species *Thermofilum**uzonense* strain 1807-2^T^. This new data expands the knowledge on physiology and diversity of this deep lineage in archaeal domain.

## Organism Information

### Classification and features

In 2008, a gray mud sample was collected from the hot spring (T 83 °C, pH 6.2) located in Orange Thermal Field, Uzon Caldera, Kamchatka, Russia (54.30 N 160.00 E). An enrichment culture was obtained using strictly anaerobic modified freshwater Pfennig medium with cellobiose and yeast extract (2 g l^−1^ and 1 g l^−1^, respectively) as substrates [8]. After 4 days of incubation at 84 °C at pH 5.8 two different types of cells – extremely thin rods and regular small cocci – were detected in the enrichment culture.

Strain 1807-2^T^ was purified by serial dilution technique on the same medium in the presence of 1/100 (v/v) *Fervidicoccus fontis* strain 1910a culture broth as it was performed for “*Thermofilum adornatus*” strain 1910b [7].

**Fig. 1:**
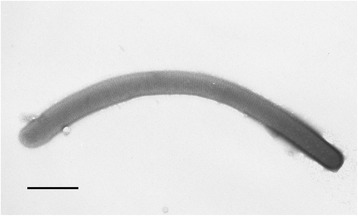
Electron micrograph of *Thermofilum uzonense* strain 1807-2^T^. Bar, 0.5 μm. Electron micrograph of negatively stained cell of *Thermofilum uzonense* strain 1807-2^T^. Cells were grown on glucose containing medium at 85 °C and incubated for two days (late exponential phase of growth). Bar, 0.5 μm

Cells of strain 1807-2^T^ were non-motile thin straight or curved filaments (Fig. 1), 0.15–0.3 μm width and 2–100 μm length.

Strain 1807-2^T^ was a hyperthermophile and obligate anaerobe. It grew optimally at 85 °C and pH 6.0–6.5 without sodium chloride in the medium. In the presence of 10 g l^−1^ NaCl growth of the strain ceased completely. Addition of at least 25 mg l^−1^ of yeast extract was mandatory. Peptone, yeast extract, starch and glucomannan were used as substrates for growth while amorphous cellulose [9] and filter paper, mannan, amorphous chitin [10], chitosan, glycerol and carbon monoxide did not support it (Table 1). Addition of 1/100 (v/v) of culture broth filtrates of other *Crenarchaeota* (*Fervidicoccus fontis*, *Desulfurococcus kamchatkensis* or *Pyrobaculum* sp.) which served, most probably, as a source of growth factors, was obligatory required for growth of strain 1807-2^T^. Filtrates could not be replaced by cell wall-fractions of these organisms, or bacterial (*Caldicellulosiruptor kronotskyensis*) culture broth filtrates.

Strain 1807-2^T^ was deposited in DSMZ (German collection of microorganisms and cell cultures) under accession number DSM 28062, and in JCM (Japan Collection of Microorganisms) under accession number JCM 19810.

**Fig. 2:**
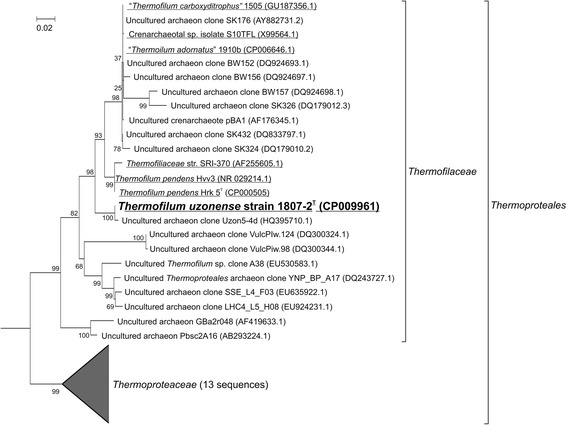
16S rRNA gene Maximal-likelihood phylogenetic tree of representatives of *Thermofilaceae* family. The tree was constructed using the Maximum Likelihood method based on the Tamura-Nei model [39]. The tree with the highest log likelihood (−5739.3765) is shown. The percentage of trees in which the associated taxa clustered together (bootstrap test of 1000 replications) is shown next to the nodes. Initial tree(s) for the heuristic search were obtained by applying the Neighbor-Joining method to a matrix of pairwise distances estimated using the Maximum Composite Likelihood (MCL) approach. A discrete Gamma distribution was used to model evolutionary rate differences among sites (4 categories (+G, parameter = 0.2411)). The tree is drawn to scale, with branch lengths measured in the number of substitutions per site. The analysis involved 38 nucleotide sequences; all were longer than 1300 nucleotides. All positions containing gaps and missing data were eliminated. There was a total of 1.227 positions in the final dataset. Evolutionary analyses were conducted in MEGA6 [40]. *Thermoproteaceae* branch includes 13 sequences (Additional file 5). Cultivated strains are underlined. *Thermofilum uzonense* strain 1807-2^T^ is in bold. Complete 16S rRNA gene of *Desulfurococcus kamchatkensis* (NC_011766.1, *Desulfurococcales* order) was chosen as an out-group. Bar, 2 substitutions per 100 nucleotides

16S rRNA gene-based phylogenetic analysis of strain 1807-2^T^ placed it into *Thermofilaceae* family being most closely related to *Thermofilum pendens* Hrk5^T^ showing 95.7 % sequence identity according to calculations, considered used substitution model (Fig. 2). For the analysis a complete 16S rRNA gene of strain 1807-2^T^ and almost complete (>1300 nt) sequences of its 50 best BLAST hits from GenBank nr/nt database, filtered through 100 % identity filter were used (final dataset included 38 sequences). 13 representative of *Thermoproteaceae *were used as an outgroup for the analysis (Additional file 5).

On 1st of May 2015 both RDP (Release 11, Update 3) and Silva (SSU r122) databases contain altogether 400 unique 16S rRNA genes of *Thermofilaceae* family clones and isolates. Most of them were partial, hence were not involved in the mentioned-above phylogenetic analysis. The analysis of distribution of their isolation sources was performed using our homemade software GetIsolationSources [11]. It searches for the GenBank accession numbers in SILVA, RDP and other databases, containing it, and extracts the information, residing under modifiers “description”, “accession”, “isolation source”, “country” and “references” from the respective GenBank records. Analysis revealed that the majority of *Thermofilaceae* family clones and pure cultures were obtained from hot springs, both terrestrial and marine. A few others were collected from various non-thermal environments. All GenBank IDs of these 16S rRNA genes are listed in Additional file 1.

## Genome sequencing information

### Genome project history

The sequencing project started in May 2013 and finished in August 2014. Due to the fact that both short and long insert libraries were used for sequencing, the obtained circular genomic contig can be considered as a finalized genome sequence. The complete genome sequence of *T. uzonense* strain 1807-2^T^ has been deposited in DDBJ/EMBL/GenBank under the accession number CP009961. Related project information and sample details have been deposited in NCBI database under accession numbers PRJNA262459 and SAMN03083278, respectively (Table 2).

**Table 1:** Classification and general features of *Thermofilum uzonense* strain1807-2^T^ [31]

MIGS ID	Property	Term	Evidence code^a^
	Current classification	Domain *Archaea*	TAS [32]
		Phylum *Crenarchaeota*	TAS [33]
		Class *Thermoprotei*	TAS [34]
		Order *Thermoproteales*	TAS [3, 4, 35–37]
		Family *Thermofilaceae*	TAS [19]
		Genus *Thermofilum*	TAS [5]
		Species *Thermofilum uzonense*	IDA
		Type strain 1807-2	IDA
	Gram stain	Not reported	
	Cell shape	Thin straight or curved rods	IDA
	Motility	Non motile	IDA
	Sporulation	Non-sporulating	IDA
	Temperature range	70–90 °C	IDA
	Optimum temperature	85 °C	IDA
	pH range; optimum	5.5–7.0; 6.0–6.5	IDA
	Carbon source	Yeast extract, peptone, starch, glucomannan	IDA
	Energy source	Yeast extract, peptone, starch, glucomannan	
MIGS-6	Habitat	Hot spring	
MIGS-6.3	Salinity	0–0.5 % NaCl (w/v). Optimum 0 %.	IDA
MIGS-22	Oxygen	Anaerobe	IDA
MIGS-15	Biotic relationship	Free living	IDA
MIGS-14	Pathogenicity	Non-pathogenic	NAS
	Biosafety level	1	NAS
	Isolation	Water/sediment of hot spring, Uzon Caldera, Kamchatka	IDA
MIGS-4	Geographic location	Uzon Caldera, Kamchatka, Far-East Russia	IDA
MIGS-5	Sample collection time	2008	IDA
MIGS-4.1 MIGS-4.2	Latitude	54 30.382	IDA
	Longitude	160 00.103	IDA
MIGS-4.3	Depth	Surface	IDA
MIGS-4.4	Altitude	663 m	IDA

^a^ Evidence codes - *IDA* inferred from direct assay, *TAS* traceable author statement (i.e., a direct report exists in the literature), *NAS* non-traceable author statement (i.e., not directly observed for the living, isolated sample, but based on a generally accepted property for the species, or anecdotal evidence). These evidence codes are from the Gene Ontology project [38]

**Table 2:** Project information and its association with MIGS version 2.0 compliance [31]

MIGS ID	Property	Term
MIGS 31	Finishing quality	Finished
MIGS-28	Libraries used	Illumina Nextera fragment library (insert mean length of 175 bp)
		PGM mate-paired library (insert mean length of 2400 bp)
MIGS 29	Sequencing platforms	Ion Torrent, Illumina, Sanger
MIGS 31.2	Fold coverage	Ion Torrent 13.6 ×
		Illumina 180.4 ×
MIGS 30	Assemblers	CLC Bio [13]
MIGS 32	Gene calling method	GeneMarKS+
	Locus Tag	MA03
	Genbank ID	CP009961
	GenBank Date of Release	April 1, 2015
	GOLD ID	Gp0108853
	BIOPROJECT	PRJNA262459
MIGS 13	Source Material Identifier	DSM 28062
	Project relevance	Evolution, Diversity

**Table 3:** Genome statistics

Attribute	Number of genes	% of Total genes^a^
Genome size (bp)	1,611,988	100.00
DNA coding^b^ (bp)	1,270,203	78.8
DNA G+C (bp)	772,789	47.9
DNA scaffolds	1	100.00
Total genes	1697	100.00
Protein coding genes	1455	85.7
RNA genes	50	3.0
Pseudo genes	192	11.3
Genes in internal clusters	ND^c^	ND^c^
Genes with function prediction	1334	78.6
Genes assigned to COGs	1389	81.9
Genes with Pfam domains	1066	62.8
Genes with signal peptides	343	20.2
Genes with transmembrane helices	129	7.6
CRISPR repeats	2	–

^a)^ The total is based on either the size of the genome in base pairs or the total number of genes in the annotated genome

^b)^ Corresponding to functional CDS. Pseudogenes are not included in this calculation

^c)^ *ND* not determined

### Growth conditions and genomic DNA preparation

*T. uzonense* strain 1807-2^T^ was grown in medium mentioned above with glucose and yeast extract (1 g l^−1^ and 0.5 g l^−1^, respectively) as substrates and in the presence of 1/100 (v/v) *Desulfurococcus kamchatkensis* strain 1221n culture broth as a source of growth factors at 85 °C during 4 days. Cell-free culture broth of *D. kamchatkensis* was obtained by filtration of grown cells through 0.22 μm filter. For that, cells of strain 1221n were grown on the same medium containing glucose and yeast extract as growth substrates at optimal conditions [12]. After cultivation, cells of strain 1807-2^T^ were disrupted with glass beads using Minilys homogenizer (Bertin technologies, France) and DNA was extracted using QIAamp DNA mini kit (Qiagen, Netherlands) according to the manufacturers’ instructions.

### Genome sequencing and assembly

For sequencing of *T. uzonense* genome both fragment and mate-paired libraries were used. Fragment library for Miseq paired-end sequencing was prepared from 50 ng genomic DNA with NexteraTM fragment library kit (Illumina, San Diego, CA, USA) according to manufacturer instructions. Analysis of library size distribution showed the mean insert size of 175 bp. Sequencing of this library using MiSeq instrument resulted in 932,095 pairs of 250 bp reads which were subjected to stringent quality and adapter trimming with the corresponding tool of CLCBio Genomics Workbench 7.5 (Qiagen, Netherlands). After trimming and filtering procedures 911,060 read pairs were used for assembly.

The mate-paired library with target insert size of 2400 bp for Ion Torrent sequencing was prepared from 3 μg of DNA with SOLiD 5500 mate-paired library kit (Life Technologies, Carlsbad, CA, USA) using Ion Torrent PGM adapters for final amplification. Library was sequenced on a 314 chip resulting in 300,306 single sequences. The internal adapter used for library construction was trimmed during import procedure to CLC Genomics Workbench. After quality and length filtering totally 197,320 read pairs were used for *de novo* assembly guidance and scaffolding (single reads were excluded from further analysis).

The initial assembly was made with CLCBio *de novo* assembler using word size of 64 and bubble size of 500 bp [13]. Since overall quality of MiSeq reads is generally much higher than PGM reads quality, the Illumina reads were used for assembly of contigs, while PGM reads used only in “guidance only” mode for de Bruijn graph ambiguities resolution. Twenty two scaffolds with L50 of 456,782 bp were obtained. After *in silico* closure of gaps with GapFiller v1.11 [14] contigs were subjected to the second round of scaffolding and gapfilling with SSPACE v.2.0 [15] and GapFiller. Finally one gapless contig of 1,610,790 bp was obtained. Assembly validation was accomplished by mapping of all high-quality MiSeq reads to the final chromosome. Analysis of the mapping revealed 3 problematic regions characterized by the enrichment of mapping conflicts and unaligned read ends. These regions were amplified by PCR and sequenced by Sanger method with ABI 3730XL DNA analyzer (Life Technologies, Carlsbad, CA, USA). Chromosome circularity was confirmed by PCR with outward-oriented primers and subsequent Sanger sequencing. After all correction procedures the length of *Thermofilum**uzonense* strain 1807-2^T^ circular chromosome was 1,611,988 bp.

### Genome annotation

Identification of genes and primary annotation was performed with PGAAP pipeline [16]. As a part of the pipeline, protein-coding genes were detected with GeneMarkS+ [17], tRNA genes were identified with tRNA-scan-SE [18] and rRNA genes were identified with blastn search against a curated NCBI rRNA reference set.

Assignment of predicted CDS to the clusters of orthologous groups (COGs) was made using BLAST [19] against the latest version of COG database [20] with maximal e-value of 10^−5^. Identification of conserved domains families was performed using HMMER hmmscan service [21]. Proteins harboring signal peptides and transmembrane helices were identified with Phobius server [22] Twin-arginine signal peptides were predicted using TatP server [23], while non-classical signal peptides were predicted using SecretomeP server [24]. Other predictions were done according to the genome annotation protocol [25].

## Genome properties

The genome of *T. uzonense* strain 1807-2^T^ consists of one circular chromosome of a total length of 1,611,988 base pairs (47.9 % G+C content). A total of 1697 genes were predicted, 1455 of which are protein-coding genes (CDS), 50 are RNA genes and 192 are pseudogenes. The genome has one ribosomal operon consisting of single copies of 16S and 23S ribosomal RNA genes, while 5S gene is located 300 kb apart from it. A search for IS elements using IS Finder database [26] showed that genome contains two IS200/IS605 family IS elements, one of which is inactive due to disrupted transposase gene. The chromosome also contains two CRISPR stretches of total length about 13 kb [27].

**Fig. 3:**
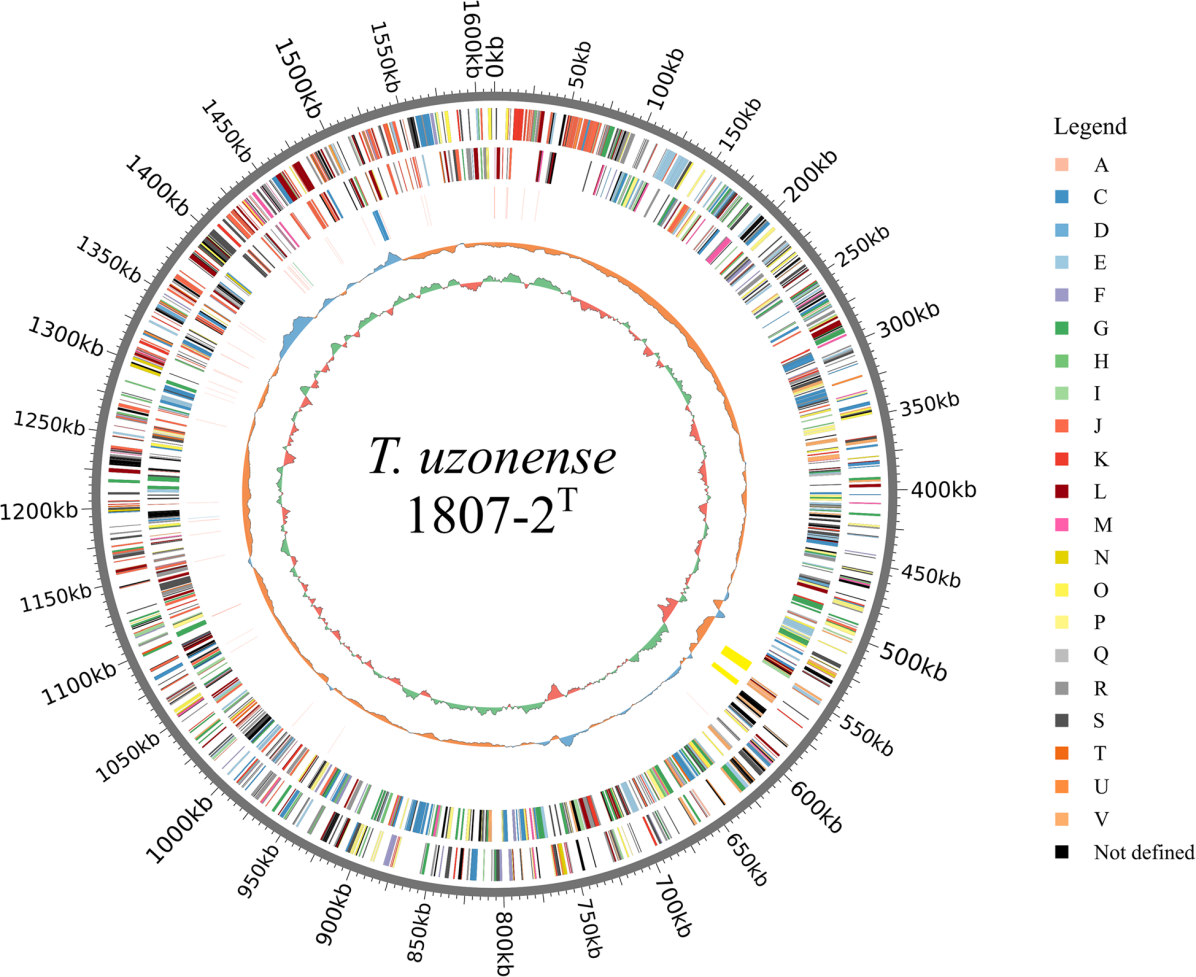
Circular map of *Thermofilum uzonense* strain1807-2^T^ generated with CIRCOS [41]. From outside to inside: positive strand CDSs colored by COG FCs (Clusters of Orthologous Genes Functional Categories), negative strand CDSs colored by COG FCs, RNA-genes and CRISPRs (tRNA – *purple*; rRNA – *blue*; riboswitches – *green*, CRISPR repeats - *yellow*), G+C-content, GC-skew. On the right – COG functional categories color codes

Approximately 70 % of CDS (1019 of 1455) were associated with Clusters of Orthologous Groups (COGs). Since the majority of individual domains of multidomain proteins were assigned to different COGs the number of COGs is higher than the number of proteins with COGs: 1389 (Table 3). The distribution of genes into COGs functional categories is presented in Table 4. Graphical map of *T. uzonense* strain 1807-2^T^ circular chromosome is presented on Fig. 3.

**Table 4:** Number of genes associated with general COG functional categories

Code	Value	% age	Description
J	146	10.0	Translation, ribosomal structure and biogenesis
A	0	0.0	RNA processing and modification
K	81	5.6	Transcription
L	81	5.6	Replication, recombination and repair
B	0	0.0	Chromatin structure and dynamics
D	4	0.3	Cell cycle control, Cell division, chromosome partitioning
V	79	5.4	Defense mechanisms
T	19	1.3	Signal transduction mechanisms
M	35	2.4	Cell wall/membrane biogenesis
N	12	0.8	Cell motility
U	13	0.9	Intracellular trafficking and secretion
O	63	4.3	Posttranslational modification, protein turnover, chaperones
C	116	8.0	Energy production and conversion
G	121	8.3	Carbohydrate transport and metabolism
E	122	8.4	Amino acid transport and metabolism
F	48	3.3	Nucleotide transport and metabolism
H	69	4.7	Coenzyme transport and metabolism
I	24	1.6	Lipid transport and metabolism
P	79	5.4	Inorganic ion transport and metabolism
Q	6	0.4	Secondary metabolites biosynthesis, transport and catabolism
R	216	14.8	General function prediction only
S	55	3.8	Function unknown
–	436	30.0	Not in COGs

The total is based on the total number of protein coding genes in the genome

## Insights from the genome sequence

Average nucleotide identity was calculated by ANI calculator [28] with default parameters. ANIs between the genomes of strain 1807-2^T^ and *T. pendens* strain HRK 5^T^ and “*T. adornatus*” strain 1910b were 85 and 82 %, respectively which is below the species border, proposed to be 95 % [29]. These results, together with the data, obtained during 16S rRNA gene-based phylogenetic analysis and phenotypic differences, support the proposal of the novel species. Genome analysis of *T. pendens* revealed a massive loss of biosynthetic pathways, supporting the observation that *T. pendens* grows only in nutrient-rich environments [30]. To check if this is a common property of *Thermofilaceae* we analyzed the presence of crenarchaeal biosynthetic COGs [30] in *in silico* translated proteomes of all members of this family with currently available genome sequences (assembly accession numbers GCA_000015225.1, GCA_000446015.1, GCA_000813245.1, GCA_000993805.1). Analysis revealed that all *Thermofilum* representatives have only 20–30 of 125 biosynthetic COGs present in almost all *Bacteria* and *Archaea* (Additional file 2). Interestingly, the number of biosynthetic protein domains which have been lost during evolution negatively correlates with genome size of *Thermofilaceae* family members (Additional file 3). Comparative analysis of COG functional groups distribution didn’t reveal any significant deviations showing general metabolic resemblance of *Thermofilaceae* (Additional file 4).

The analysis of *T. pendens* genome [30] revealed the possibility of growing on various polysaccharides including starch and cellulose, however none of them were tested so far. *T. uzonense* is able to grow on starch and glucomannan, and the genes encoding corresponding glycosidases were found in its genome. In total, 17 genes are predicted to encode various glycosidases. Two of them are predicted to be extracellular: MA03_06285 (GH57) and MA03_03765 (GH3). The first one is putative alpha-amylase, most probably responsible for extracellular starch hydrolysis, while the activity of the second one is not particularly obvious, but most likely, is a beta-glucosidase. Intracellular utilization of starch hydrolysis products could occur by means of four GH57 family glycosidases: MA03_00470, MA03_00770, MA03_05655 and one GH13 maltogenic amylase MA03_06280. Final intracellular hydrolysis of maltose could be catalyzed by alpha-glucosidases MA03_03630 (GH4) and MA03_04210 (GH122). Glycosidases MA03_06285 and MA03_06280 are localized in a gene cluster together with transporters MA03_06265 (MalG) and MA03_06270 (MalF) and maltooligosaccharide-binding proteins MA03_06275 and MA03_06290 indicating their synergetic action. Enzymes presumably involved in glucomannan hydrolysis are MA03_02580 (DUF377 domain-containing putative glycosidase), MA03_03185 (GH38), MA03_04200 (GH1), MA03_02300 (GH113). All predictions of their localization (SignalP, TatP, SecP) did not reveal any signatures of extracellular proteins, indicating rather unusual motifs, recognized by signal peptidases, or another modifications in their secretion system than the intracellular state of all these enzymes. Interestingly, putative glycosidase MA03_02580 is located in the cluster of ABC and MFS families of putative sugar transporters and solute-binding proteins: MA03_2555-2575 and MA03_2585 indicating their possible co-regulation.

Two genes encoding proteins MA03_04265 and MA03_06125 are homologous to NAD-dependant oxidoreductases, some of which could be involved in degradation of glycosidic bonds in polysaccharides (GH109); however their function in *T. uzonense* is unclear.

Apart from glycoside hydrolases two genes encoding carbohydrate esterases (CEs) were also found: MA03_03235 (CE14) family and MA03_04280 (CE9). Characterized enzymes of these families are predicted to be involved in metabolism of N-acetyl-glucosamines and relative compounds.

The genome contains more than 40 genes encoding peptidases of different families. Ten of them are predicted to be extracellular and four possibly are responsible for the hydrolysis of peptides during growth on peptone: subtilase (S08A family) MA03_03015, archaeal serine endopeptidase MA03_01720, thermopsin (A5 family) MA03_03850 and metalloendopeptidase (M48B family) MA03_08235.

## Conclusions

Analysis of complete genome sequence of strain 1807-2^T^ indicates that it can be proposed as a novel species *Thermofilum**uzonense* strain 1807-2^T^*.* The majority of *T. uzonense* CDSs have homologs in the genomes of other *Thermofilum* members, and their distribution among COGs functional categories shows high level of metabolic homogeneity. As it was found for other *Thermofilum* representatives, massive reduction in number of proteins, contributing to biosynthetic pathways [30], has occurred in *T. uzonense* genome, hence, like other members of the family, its lifestyle could be characterized as opportunistic heterotroph, growing in nutrient-rich environments.

Apart from peptidic substrates, *T. uzonense* is able to grow on α-linked (starch) and β-linked (glucomannan) polysaccharides. While this capability was speculated in *T. pendens* genome paper [30], until now, it was never proven experimentally. Comparative analysis of all available *Thermofilum* genomes revealed two genes coding alpha-glucosidase of GH122 family and putative carbohydrate esterase of CE4 family, were present exclusively in genome of *T. uzonense*. Another two, coding extracellular putative amylase of GH57 family and putative endomannanase of GH113 family were found only in *T. uzonense* and “*T. adornatus*” genomes. These results suggest that the ability to hydrolyze various polysaccharides could be characteristic for *Thermofilum* representatives with some substrate-specific peculiarities of each individual strain. This allows one to conclude that *Thermofilum* members should not be regarded solely as consumers of simple organic substrates but also as primary destructors of the complex organic matter which occurs in their environments.

## Taxonomic and nomenclatural proposals

### Description of *Thermofilum uzonense* sp. nov.

*Thermofilum uzonense* (u.zo.nen’se N.L. neut. adj. *uzonense*, pertaining to the Uzon Caldera, Kamchatka, Far-East Russia, from where the type strain was isolated).

Cells are non-motile thin straight or curved filaments, 0.15–0.3 μm in width and 2–100 μm in length. Strict anaerobe. Hyperthermophile growing optimally at 85 °C and pH 6.0–6.5 in freshwater medium. Utilizes starch, glucomannan, peptone and yeast extract as the substrates. Amorphous cellulose, filter paper, mannan, amorphous chitin, glycerol and carbon monoxide do not support growth. Yeast extract and culture broth filtrate of other *Crenarchaeota* are required for growth. The type strain is 1807-2^T^ (= DSM 28062^T^ = JCM 19810^T^), was isolated from a mud sample of Uzon Caldera, Kamchatka (Russia). Genome size of the type strain is 1.6 Mb. The G+C content of DNA is 47.9 mol %. The genome sequence of the strain has been deposited in DDBJ/EMBL/GenBank under the accession number CP009961.

### Emended description of the genus *Thermofilum*

The description is based on that provided by Zillig and colleagues [5], with the following amendments. The genus contains slightly or moderately acidophilic species utilizing peptides and polysaccharides and requiring polar component lipid from *Thermoproteus tenax* or culture broth of other *Crenarchaeota* for growth. Type species: *Thermofilum pendens**.*

## Additional files

Additional file 1:
***Thermofilaceae*** **representatives. List of all, according to the latest releases of RDP (Release 11, Update 3) and Silva (SSU r122) databases,** ***Thermofilaceae*** **isolates and clones and their isolation sources.** (XLSX 19 kb) Additional file 2:
**Biosynthetic COGs. List of biosynthetic COGs among** ***Thermofilum*** **representatives with sequenced genomes.** (XLSX 16 kb) Additional file 3:
**Biosynthetic COGs and genome size.** Correlation between the number of proteins, presumably involved in anabolism, and genome size. (JPG 32 kb) Additional file 4:
**COGs distribution by functional groups.** Distribution of *Thermofilum* representatives COGs by functional groups (XLSX 13 kb) Additional file 5:
***Thermoproteaceae*** **sequences. List of 13** ***Thermoproteaceae*** **sequences, collapsed in a triangle on the Fig.** **2.** (TXT 601 bytes)

## Abbreviations

ANI: Average nucleotide identity
COG: Clusters of orthologous genes
DSMZ: German collection of microorganisms and cell cultures
FC: Functional categories
GH: Glycoside hydrolase
JCM: Japan collection of microorganisms
PGM: Ion Torrent personal genome machine
RDP: Ribosomal Database Project
