# Supplementary figures and images for: Complete genome sequence of and proposal of Thermofilum uzonense sp. nov. a novel hyperthermophilic crenarchaeon and emended description of the genus Thermofilum
Source: Stand Genomic Sci. 2015 Dec 9;10:122. doi: 10.1186/s40793-015-0105-y (PMC4673724; doi:10.1186/s40793-015-0105-y)

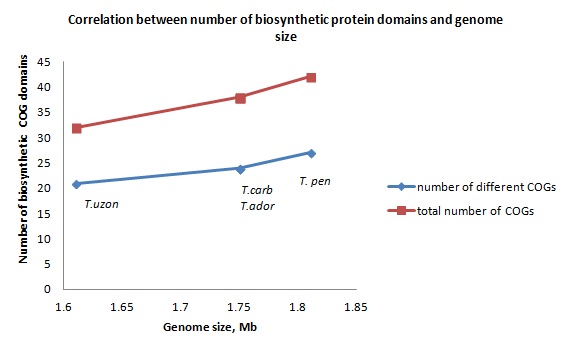

Supplement: Additional file 3: — Biosynthetic COGs and genome size. Correlation between the number of proteins, presumably involved in anabolism, and genome size. (JPG 32 kb) [file 40793_2015_105_MOESM3_ESM.jpg]
